# Functional Characterization of the Gustatory Sensilla of Tarsi of the Female Polyphagous Moth *Spodoptera littoralis*

**DOI:** 10.3389/fphys.2018.01606

**Published:** 2018-11-14

**Authors:** Mervat A. Seada, Rickard Ignell, Abdel Naieem Al Assiuty, Peter Anderson

**Affiliations:** ^1^Unit of Chemical Ecology, Department of Plant Protection Biology, Swedish University of Agricultural Sciences, Alnarp, Sweden; ^2^Department of Zoology, Tanta University, Tanta, Egypt

**Keywords:** contact chemosensilla, gustatory receptor neurons, taste encoding, electrophysiology, Lepidoptera, tarsomere

## Abstract

Contact chemoreception is crucial for host plant choice selection in insects and is guided by input from gustatory receptor neurons, GRNs, housed in gustatory sensilla. In this study, the morphology and response spectra of individual tarsal sensilla on the fifth tarsomere of females of the moth *Spodoptera littoralis* were investigated. Two distinct morphological types of gustatory sensilla, TI and TII, were identified. Extracellular electrophysiological recordings were performed on each sensillum type using three sugars, two bitter substances and salt. Three distinct functional classes (TIα, TIβ, TII) were characterized, using cluster analysis based on the response spectra of three of the four responding GRNs. While each functional type of sensillum housed GRNs responding to salt, sugars and bitter compounds, the identity of these cells differed among the functional classes. Interestingly, an interaction between the GRNs responding to sugar and caffeine was found in both TIβ and TII sensilla, when binary mixtures were tested. This study provides a functional screening of the tarsal gustatory sensilla, showing a differentiation between sensilla on the tarsi of *S. littoralis*, providing the female moth with information that can facilitate host plant choice decisions.

## Introduction

Host plant selection in phytophagous insects involves orientation, landing and ultimately contact evaluation of potential host plants (Schoonhoven et al., 2005). Contact chemical cues are crucial for insect herbivores during the final phase of the process of host recognition, where they provide vital information for the final acceptance or rejection of a plant for feeding or oviposition. To assess the suitability of host plants, phytophagous insects use both primary and secondary metabolites on the plants that act as stimulants and deterrents (Renwick, 1989). These metabolites are detected by gustatory receptor neurons (GRNs) housed in sensilla on the antennae, mouthparts, tarsi and ovipositor of insects (Chapman, 2003). Functional characterisation of the GRNs in female insects could lead to an increased understanding of the mechanisms underlying the acceptance and rejection of resources required for survival and reproduction, and thereby the development of novel control strategies.

Sensory input from insect tarsal GRNs responding to sugars and secondary metabolites has been shown to be important for the evaluation of potential feeding and oviposition sites (Chapman, 2003). Sugars, including sucrose, glucose, and fructose, are phagostimulants that are present in floral and extrafloral nectar, two important food sources for numerous insects, including moths (Baker and Baker, 1983). In addition, the presence of sugars on the surface of the green parts of a plant may indicate the nutritional quality of the plant (Muller and Riederer, 2005). In contrast, secondary metabolites, which have a bitter taste, present in nectar and on the plant tissue, in many cases signal that the food is noxious or unpalatable for the insect (Adler, 2000). In humans, bitter taste is defined as a sensation associated with the perception of potentially toxic compounds such as alkaloids, which induce innate aversive reactions (Ventura and Worobey, 2013).

The Egyptian cotton leafworm, *Spodoptera littoralis* (Lepidoptera: Noctuidae) is a polyphagous herbivore. Although it can accept many plants as a host, it discriminates between host plants of different quality (Anderson et al., 1993; Thöming et al., 2013; Zakir et al., 2013a; Proffit et al., 2015). The selection of a suitable host plant in *S. littoralis* is shown to be guided by volatile cues, but there are also strong indications that contact cues from the plants, in combination with volatile cues, are important (Anderson and Alborn, 1999; Zakir et al., 2013b). Early electrophysiological observations from gustatory sensilla in both larvae and adult females of *S. littoralis* have demonstrated responses to sugars and secondary metabolites (Blaney and Simmonds, 1990; Simmonds et al., 1992). In addition, responses to salts and sugars have been monitored on GRNs on the antenna of female *S. littoralis* (Popescu et al., 2013). It has also been shown that GRNs on the ovipositor of females respond to salt and sugars (Seada et al., 2015). However, our knowledge about the peripheral sensory characterization of the tarsal GRNs of the female *S. littoralis* according to sensillum type and location is limited.

In this study, we characterize all chemosensory sensilla found on the ventral surface of the fifth tarsomere of the prothoracic legs of the female *S. littoralis*, as this part of the tarsus is among the first to contact the plant when the moth alights, and thus potentially is important during host plant selection for feeding and oviposition. The main aim of the present study was to morphologically and functionally characterize these sensilla, and thereby provide an increased resolution on how individual sensory neurons encode basic compounds involved in host plant recognition, including salt, sugars and two bitter substances, caffeine and quinine. Caffeine and quinine are alkaloid secondary metabolites that have been used to analyze the behavioral and functional responses of the insect gustatory sensilla to bitter compounds in a number of studies (Meunier et al., 2003; Glendinning et al., 2006; Jørgensen et al., 2007). Another aim was to study the interaction between the sugar and deterrent gustatory neurons of the tarsal gustatory sensilla on the peripheral level by combinations of sucrose and caffeine, to access how such conflicting information is encoded at the peripheral level.

## Materials and Methods

### Insects

The *S. littoralis* used in the experiments originated from a laboratory culture initiated in 2007 with wild-caught moths from Egypt. Field collected moths from Egypt have been introduced into the culture on a yearly basis. Larvae were reared on an artificial diet according to Hinks and Byers (1976), except that potatoes were used instead of beans. Pupae were collected, sexed and then kept separated. For all experiments, 2- to 3-day old females were used. All developmental stages were kept at 25°C, 70% relative humidity, and at a light: dark cycle of 16: 8 h.

### Light and Scanning Electron Microscopy

For light microscopy, the tibiae with tarsi of female moths were dissected from the prothoracic legs, and then the fifth tarsomeres were mounted onto a microscope slide with a piece of double-sided sticky tape. The fifth tarsomere with their sensilla was then examined under a Nikon Eclipse (E600FN; Nikon Instruments Europe BV, Netherlands) microscope. For scanning electron microscopy (SEM), the prothoracic tibiae of female moths were excised using fine scissors and immersed in 70% ethanol overnight at 4°C. Specimens were then dehydrated in 80, 90, and 100% ethanol, mounted on SEM stubs, and sputter coated with gold-palladium (3:2) in a JEOL ion sputter JFC-1100. The specimens were visualized using a scanning electron microscope (LEO 435 VP, United Kingdom).

### Electrophysiological Recordings

To functionally characterize the gustatory sensilla, moths were restrained in a holder made of a 1 ml plastic, disposable, pipette tip with one of their prothoracic legs protruding from the tip of the pipette. To prevent movement of the moth, the exposed head and prothorax were covered with wax. The mounted insect was then fixed onto a microscope slide, and the leg was secured onto an elevated cover slip with a piece of double-sided sticky tape. A tungsten wire (diameter 0.12 mm, Harvard Apparatus Ltd., Edenbridge, United Kingdom), serving as a reference electrode, was inserted into the abdomen of the insect and fixed with wax. Once mounted, the moth was placed under a Nikon Eclipse microscope so that the sensilla were visible at high magnification (750×).

Electrophysiological recordings were performed using the tip recording technique (Hodgson et al., 1955). Electrodes, with a tip diameter of ∼20 μm were manufactured from borosilicate glass capillaries (1.0 mm outer diameter × 0.75 mm inner diameter, Harvard apparatus, TW100-3, United States), using a vertical electrode puller (PP830; Narishige, Japan). The recording electrode was filled with a test or a control stimulus just prior to the start of a recording. The electrode was then connected to a taste probe (Syntech, Kirchzarten, Germany), which permitted reliable AC recordings from the GRNs housed in the individual sensilla. The taste probe was connected to an amplifier (Taste Probe DT-02, Syntech) with an automatic compensation of the offset (Marion-Poll and van der Pers, 1996). Electrical signals were further amplified and filtered (bass band filter: 100–1000 Hz) with an analog to digital signal converter (IDAC, Syntech), which was connected to a PC computer for signal recording and visualization. The recording electrode was placed over an individual sensillum using a micromanipulator (DC-3K, Märzhäuser Wetzlar GmbH & Co., KG, Wetzlar, Germany). Stimulation was made for 3-to-5 s with an inter-stimulus interval of approximately 6 min to avoid adaptation. Data were recorded from ∼50 females to accumulate the complete data set from all sensilla, with 3–6 sensilla tested per individual.

To functionally characterize the gustatory sensilla present on the fifth tarsomere of females, we tested sucrose, fructose and glucose at a concentration of 10 mM; caffeine and quinine at concentrations of 1.0 and 0.1 mM, respectively. The reason for using a lower concentration of quinine was that higher concentrations had detrimental effects on the sensory neurons. The sugars and the bitter compounds were dissolved in a 10 mM NaCl solution in double-distilled water. A solution of 10 mM NaCl served as the control stimulus. All compounds used in this study were of analytical grade (Sigma-Aldrich Sweden AB, Stockholm, Sweden). Dose-response recordings were performed on sensilla from the different functional classes identified on the tarsi. For these experiments, sugars were tested at 0.1, 1.0, 10, and 100 mM, caffeine at 0.1, 1.0, and 10 mM, quinine at 0.01, 0.1, and 1.0 mM, and NaCl at 1.0, 10, 100, and 1000 mM. To investigate peripheral interactions between potential stimulant and deterrent neurons housed within the two morphological types of the tarsal sensilla, responses of the most active sensilla (TIβ1-3, and TII1, TII2, TII6) to single compounds of 10 mM sucrose, 1.0 mM caffeine, 10 mM caffeine and mixtures of 10 mM sucrose and 1.0 mM or 10 mM caffeine were recorded. The experiment was replicated 7–10 times. All tastants and control solutions were prepared fresh every second week and stored at 4°C. Electrophysiological responses were recorded by counting the number of action potentials (spikes) in the first second after stimulus onset.

### Analysis

In the electrophysiological recordings, extracellular spikes of three to four GRNs could be distinguished in each sensillum, and were sorted manually according to differences in amplitudes and waveforms. Based on spike amplitude these are referred to as “N1,” “N2,” “N3,” and “N4,” with the N1 having the largest spike amplitude and N4 the smallest. To determine whether individual GRNs housed in gustatory sensilla on the prothoracic fifth tarsomere have functionally different response profiles, we conducted a complete linkage cluster analysis with squared Euclidean distances. For this, the mean firing rate of each GRN housed in the twelve ventral sensilla on the fifth tarsomere of the female moth were used to generate a dendrogram (Minitab Release 14.12.0, Minitab Inc., State College, United States). The dendrogram generated is representative of the relationships between each GRN response profile to all of the tested stimuli in a multidimensional space. GRNs found to cluster together are therefore considered to be a functional class. Analysis of variance (ANOVA) using the generalized linear model (GLM) procedure was performed to test the effect of concentrations, the types of sugar and the interaction between them on the firing activity of the sugar responsive neurons associated with the two distinct sensillum types TI and TII of the female moth. When significant effects were detected, multiple comparisons (Bonferroni test) were tested (Minitab Release 18). Furthermore, comparisons between firing rates of sugar- or bitter-sensitive GRNs of TII sensilla of the female with individual tastants and mixtures of them were done by one-way ANOVA followed with a Tukey-Kramer multiple comparisons test.

## Results

### Morphology of the Fifth Tarsomere Gustatory Sensilla

**FIGURE 1:**
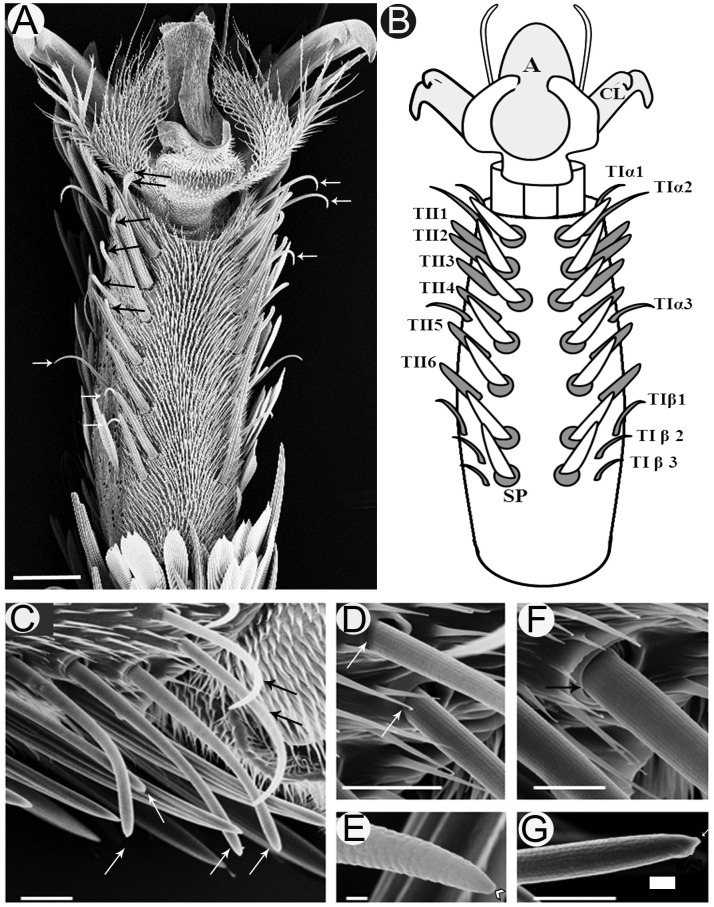
Scanning electron micrographs of the ventral surface of the fifth tarsomere of female *S. littoralis* showing two parallel rows of *sensilla chaetica*. **(A)** These sensilla may be distinguished by their location and morphology as TIα (upper white arrows), TII (black arrows), and TIβ (lower white arrows) sensilla. **(B)** Schematic diagram illustrating the different sensillum types and numbers: TIα1-3, TII1-6, and TIβ1-3. **(C)** A magnified view of the anterior-lateral part of the fifth tarsomere showing the TIα1-2 (black arrows) and TII1-4 sensilla (white arrows). **(D)** The basal socket structure of the TI sensillum (white arrows), and **(E)** its fine tapering uniporous tip (black arrow). **(F)** The basal folded socket structure of the TII sensillum (black arrow), and **(G)** its blunt uniporous tip (white arrow). Notice the swollen knob close to the sensillum pore (arrow head). A, arolium; CL, claw; SP, spine. Scale bars: 50 μm in A*;* 20 μm in C; 1 μm in E; and 10 μm in D, F, and G.

Investigation of the fifth tarsomere of the female *S. littoralis* with light microscope revealed that the prothoracic legs are covered with scales except on the ventral surface, which carries two symmetrical rows of seven spines centrally (Figures 1A,B). Lateral to the spines, there are two parallel rows of *sensilla chaetica* (Figure 1A). These sensilla are differentiated into two morphological types: TI and TII (Figures 1B,C). TI sensilla are thin, with prominent basal articulating sockets, and a distinct apical pore visible at high magnification (Figures 1D,E). TII sensilla, on the other hand, are thick, with prominent folded basal articulating sockets, and a swollen knob found beside the apical pore (Figures 1F,G). The cuticle of the hair shaft of both T1 and TII sensilla has an outer cuticular annular ornamentation (Figures 1D,E).

Examination of the right foreleg showed that there are six (in ∼90% of tested insects) to eight (in ∼10% of tested insects) (*n* = 100) TI sensilla at each ventro-lateral side of the fifth tarsomere. For insects with the most common number (six sensilla), these sensilla were distributed as follows; two distal TI sensilla at the tip, one middle TI sensillum between TII4 and TII5, and three proximal TI sensilla at the base of the tarsomere (Figures 1A,B). Similarly, six to eight TII-sensilla, with the most common number being six (TII1-6), are distributed in the middle region of the fifth tarsomere, each of which is closely associated with a stout spine at the internal margins of the tarsomere (Figures 1A,B). In the electrophysiology study only females with twelve sensilla (six of each type) on the fifth tarsomere were tested.

### Functional Classification

**FIGURE 2:**
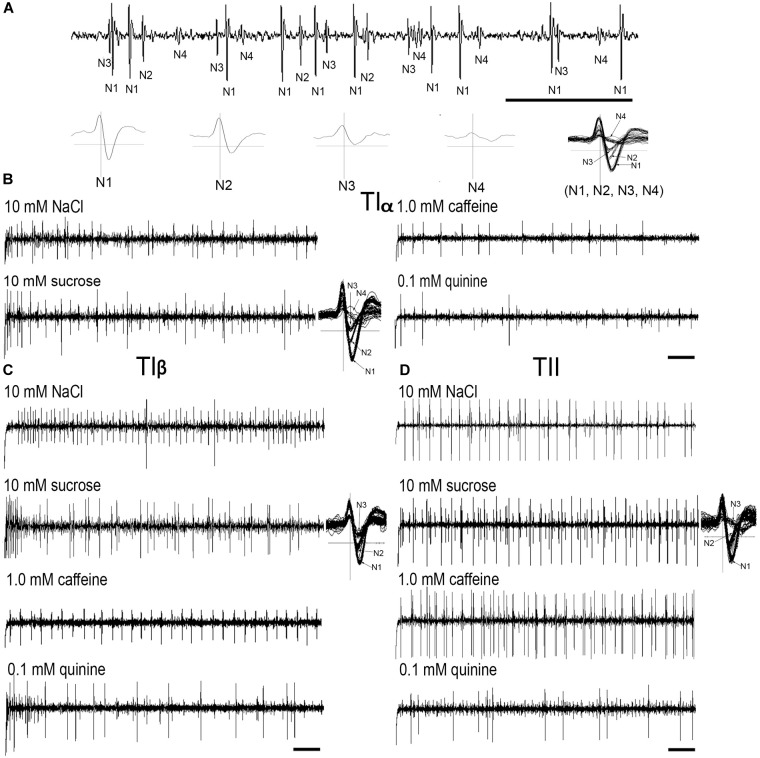
Typical recordings from the tarsal gustatory sensilla showing spike trains evoked in the tarsal gustatory receptor neurons (GRNs) of TIα, TIβ, and TII sensilla associated with the fifth tarsomere of female *S. littoralis*. **(A)** Electrical identification of four GRNs according to spike amplitudes in response to the electrolyte (up) and the wave forms of (N1-4) separately and all activated neurons (N1, N2, N3 and N4) together (down). **(B–D)** Example recordings from TIα, TIβ and TII sensilla to 10 mM NaCl, 10 mM sucrose, 1.0 mM caffeine and 0.1 mM quinine, respectively and examples of spike analyses of the responsive GRNs with 10 mM sucrose (Scale bars: 100 ms in A; and 200 ms in **B–D**).

**FIGURE 3:**
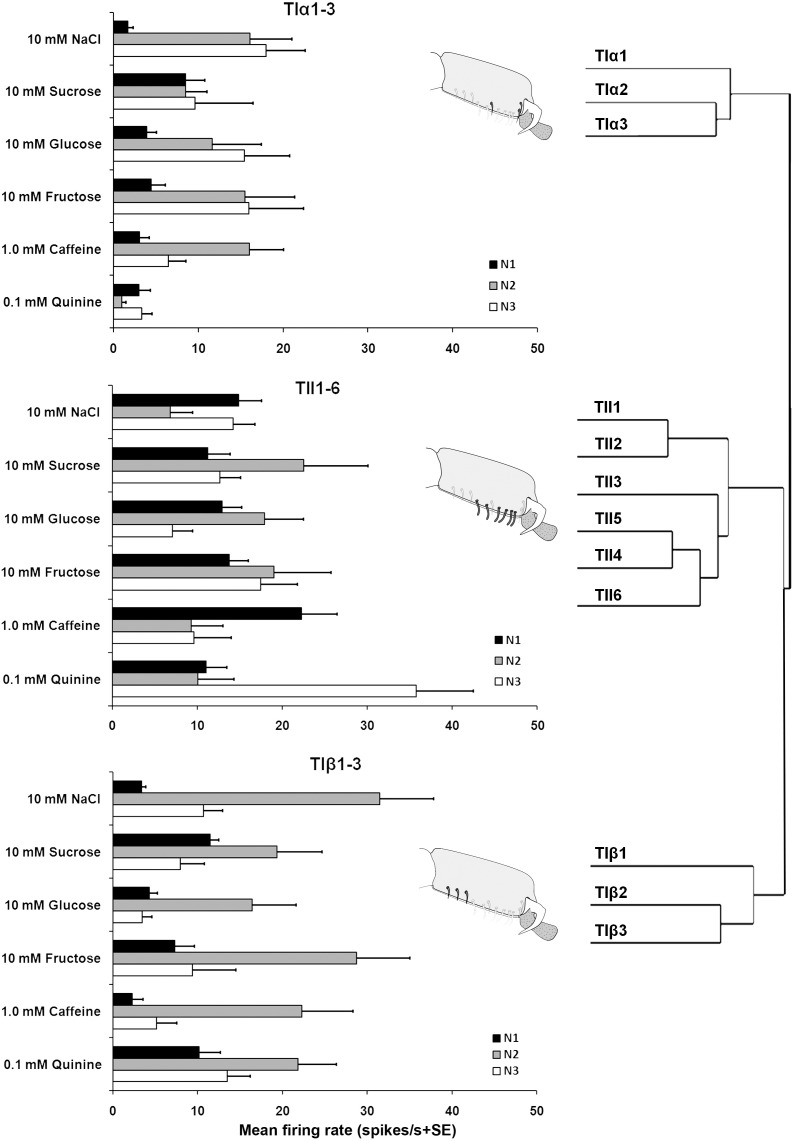
Electrophysiological responses (mean ± SE spikes/ s) of three GRNs (N1, N2, and N3) within each functional class of the gustatory sensilla associated with the fifth tarsomere of female *S. littoralis*to10 mM NaCl, 10 mM sucrose, 10 mM fructose, 10 mM glucose, 1.0 mM caffeine, and 0.1 mM quinine. At the right, the cluster analysis based on the overall responses against the screening tastants of the GRNs is shown. Errors bars represent standard error. *n* = 8–15.

Electrophysiological recordings were made from 180 *s. chaetica* of ∼50 *S. littoralis* female moths, showing responses from three to four distinguishable GRNs, N1-N4 (Table 1 and Figure 2A). Since the N4 neuron could not consistently be identified in all traces due to high noise levels, the responses of this neuronal class were not included in the analysis. We found that spike amplitudes of all firing GRNs, within a single sensillum, were consistently different compared to each other and stable across preparations. No apparent change in spike amplitude was observed at increasing concentrations of stimuli. In general, the N1-N3 GRNs exhibited excitatory tonic or phasic-tonic neuronal responses of variable magnitude in response to the tested stimuli (Figure 2). However, in a few cases inhibitory responses were also recorded. The complete linkage cluster analysis revealed three distinct functional sensillum types, TIα, TIβ, and TII, based on the response spectra and sensitivity of the N1, N2, and N3 neurons to the stimulants tested (Figures 2B,D, 3).

**Table 1:** Responses of three gustatory receptor neurons (N1-N3) of TIα, TIβ, and TII sensilla on the fifth tarsomere of female *S. littoralis* according to spike amplitudes in response to 10 mM NaCl, 10 mM sucrose, 1.0 mM caffeine and 0.1 mM quinine, respectively. Activation of each neuron is denoted by (+) and inhibition by (−).

Chemical ligands	Responding GRNs		
	TIα-sensillum	TIβ-sensillum	TII-sensillum
−10 mM NaCl	+N2	+N2	+N1
−10 mM Sugars	+N1	+N1	+N2
−1.0 mM Caffeine	−N3	−N3	+N1
−0.1 mM Quinine	−N2	+N1	−N1
	−N3		+N3

**FIGURE 4:**
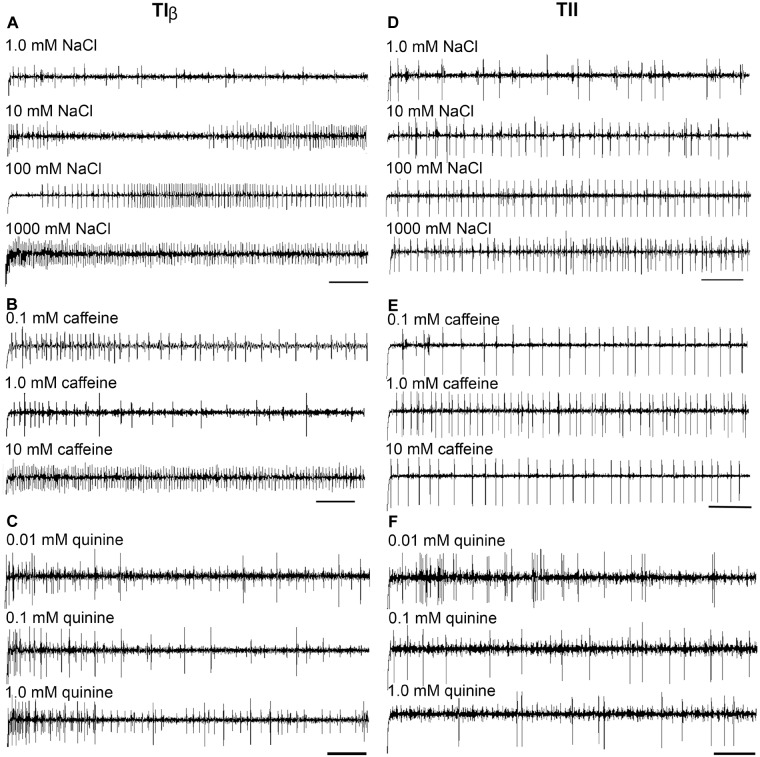
Dose–response recordings evoked by salt and bitter compounds in the TIβ, and TII gustatory sensilla associated with the fifth tarsomere of female *S. littoralis*. **(A–C)** Typical recordings from TIβ sensilla to gradient concentrations of NaCl (1.0–1000 mM); caffeine (0.1–10 mM), and quinine (0.01–1.0 mM). Whereas, the dose responses properties of TII sensilla against the same tastants are represented in **(D–F)**. The bitter compounds were dissolved in 10 mM NaCl as an electrolyte. The time panel of each trace is 2 s (Scale bars: 200 ms).

**FIGURE 5:**
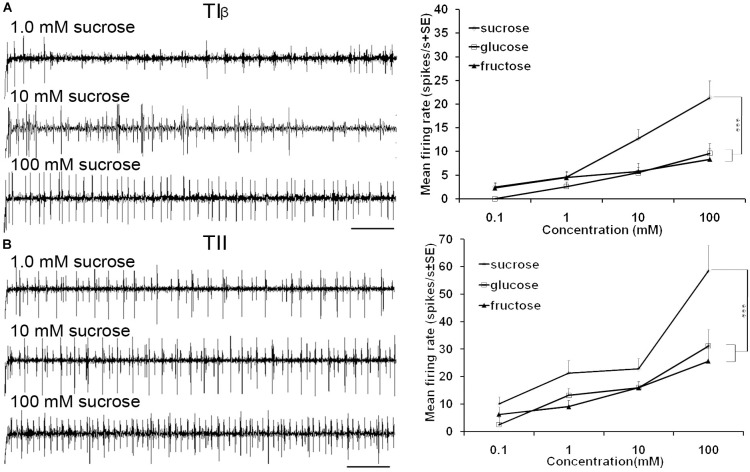
Dose-response recordings of TIβ, and TII tarsal taste sensilla associated with fifth tarsomere of female *S. littoralis* to (0.1–100 mM) sucrose, fructose, and glucose. Response properties of TIβ taste sensilla (N1-Neuron) **(A)** and TII sensilla (N2-Neuron) **(B)**. Scale bar is 200 ms (*n* = 8). Errors bars represent standard error. Asterisks indicate a significant difference between stimuli (*p* < 0.001).

Stimulation of TIα and TIβ with 10 mM NaCl showed a similar characteristic firing pattern in the N2-neuron and to sugars in the N1-neuron, but with lower firing frequencies in the TIα-sensilla (Figures 2B,C, 3, 4A, 5A). The opposite response pattern was found in the TII sensilla, where NaCl elicited a tonic firing in the N1-neuron and sugars in the N2-neuron (Figures 2D, 3, 4D, 5B). In the TI sensilla, an immediate activation at the time of contact was found in approximately 50% of the stimulated sensilla, while a delayed onset of response was found in the remaining half of the sensilla tested, with an individual variation in latency time of ∼200 to 2500 ms. Increasing concentrations of NaCl increased the firing rates of the N2-neuron associated with TIβ sensilla and reduced the latency period of excitation (Figure 4A). Recordings from TIα sensilla were often noisy, which prevented complete dose response analysis. Thus, only dose-response analyses obtained from TIβ and TII sensilla are presented (Figures 4A–F, 5A,B).

In both types of TI sensilla, we found that caffeine inhibited the response of the N3-neuron (Figures 2B,C, 3). The TII sensilla differed from TIα and TIβ sensilla in that caffeine elicited a moderate increase in the response of the N1-neuron (Figures 2D, 4E). In about one third of the recordings from the TII sensilla, a dose-dependent increase in excitation to caffeine was observed in the N2-neuron (data not shown). The other secondary metabolite, quinine, elicited different responses in all three types of sensilla. In the TIα sensilla, an inhibition of the N2- and N3-neurons was recorded (Figures 2B, 3), while in the TIβ sensilla a phasic activation of the N1-neuron was elicited during the first 1000 ms of stimulation and N2-neuron during the first 100–150 ms of stimulation before deactivation (Figures 2C, 3, 4C). At the highest concentration of quinine tested, a phasic tonic response was also exhibited in the N2-neuron (Figure 4C). In TII sensilla, quinine evoked an inhibition in the activity of the N1-neuron, and a tonic excitation in the N3-neuron (Figures 2D, 3). Increasing concentrations of quinine reduced the firing activity of the N1-neuron within the TII sensilla, accompanied with irregular pausing periods followed with burst firing (Figure 4F). In some recordings, high concentrations of quinine totally inhibited the responsive GRNs, accompanied with reductions of the spike amplitudes of N1-neurons, which might be due to a damaging effect of the high concentration of quinine.

Stimulation of TIα and TIβ sensilla with the three tested sugars evoked a clear response in the N1 neuron (Figures 2B, 5A). In TIβ sensilla, the sugar type (GLM analysis, *F* = 15.24, df = 2, *P* < 0.0001), concentration (GLM analysis, *F* = 24.62, df = 3, *P* < 0.0001) and the interaction between them (GLM analysis, *F* = 2.38, df = 6, *P* = 0.004) had a significant effect on the firing rate of the N1 neuron. A stronger phasic-tonic response was recorded for sucrose than for fructose and glucose (Bonferroni test: *t* = 4.3, *P* < 0.001 and *t* = 5.2, *P* < 0.001; respectively) (Figure 5A). However, no difference in firing activity of the N1 neuron of TIβ sensilla between fructose and glucose was recorded (Bonferroni test: *t* = −0.86, *P* > 0.05).

In the TII sensilla, the sugar sensitive N2 neuron showed higher firing frequencies than the sugar sensitive N1 neuron in the TI sensilla for the three sugars tested (Figures 2D, 5B). Similar to the TIβ sensilla, concentrations (GLM analysis, *F* = 16.56, df = 3, *P* < 0.0001), sugar types (GLM analysis, *F* = 7.63, df = 2, *P* = 0.001) and the interaction between them (GLM analysis, *F* = 3.45, df = 3, *P* = 0.025) had significant effects on the firing rate of the N2 neuron. Also in the TII sensilla, higher responses were found to sucrose than to the other two tested sugars (Bonferroni test: *t* = 3.01, *P* < 0.01 and *t* = 3.59, *P* < 0.001; respectively) while no difference in the firing activity in response to fructose or glucose was found (Bonferroni test: *t* = −0.48, *P* > 0.05). The highest concentration of sugars (1000 mM) was excluded as the noise ratio was very high and spikes resolution was difficult. This was probably due to the high viscosity of the tested solution.

### Peripheral Interaction Responses to Binary Mixtures of Sucrose and Caffeine

**FIGURE 6:**
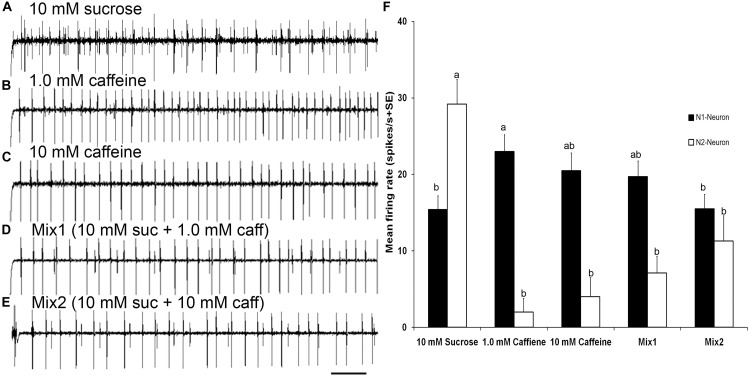
Responses to sucrose and mixtures of sucrose and caffeine **(A–E)** Temporal responses of TII tarsal gustatory sensilla of fifth tarsomere of female *S. littoralis* to sucrose, caffeine, and mixtures of sucrose and caffeine. N2-Neuron activated with sucrose, N1-Neuron activated with caffeine, while mutual inhibition of both neurons with mixtures of sucrose and caffeine were recorded. **(F)** Average responses (imp/s) of 18 sensilla of TII when stimulating with 10 mM sucrose, 1.0 mM caffeine, 10 mM caffeine, and mixtures of 10 mM sucrose and 1.0 mM or 10 mM of caffeine. There was a mutual inhibition of both sugar and deterrent responsive GRNs in TII sensilla. Scale bar is 200 ms (*n* = 7–10). Errors bars represent standard error. Different letters means significant difference (*p* < 0.05) between responses of N1 or N2 Neurons to the stimuli.

Stimulation of TII sensilla with binary mixtures of sucrose and caffeine revealed an inhibition of the sugar sensitive N2-neuron (Figures 6A–F). The first binary mixture (mix1) of 10 mM sucrose and 1 mM caffeine evoked a significantly lower firing rate of the sugar sensitive N2-neuron compared with its activity with 10 mM sucrose alone (one way ANOVA, *t* = 18.43, *P* < 0.0001). Similarly, the second binary mixture (mix2) of 10 mM sucrose and 10 mM caffeine evoked a significant deactivation of the sugar sensitive N2 neuron compared with its activity with 10 mM sucrose alone (one way ANOVA, *t* = −5.38, *P* < 0.0001). No difference in the firing rate of the bitter sensitive N1 neuron was observed when compared with its activity with 1 mM (one way ANOVA, *t* = −0.57, *P* = 0.993) and 10 mM caffeine alone (one way ANOVA, *t* = −1.88, *P* = 0.423). Stimulation of TIβ sensilla with mixtures of sucrose and caffeine also evoked an inhibition of the sugar neuron (data not presented).

## Discussion

Two morphological types of gustatory sensilla, TI and TII, are arranged in two parallel rows aligned with two symmetrical rows of seven spines centrally. Similar distribution of tarsal sensilla has been found in females in other Lepidopteran species. For example, the fifth tarsomere of female European corn borer, *Ostrinia nubilalis* have two ventral rows of about five contact chemoreceptors disposed in parallel (Marion-Poll et al., 1992). Calas et al. (2009) also described that the ventral surface of the fifth tarsomere of female *Mnesampela privata* (Lepidoptera: Geometridae) bears two parallel rows of up to eight sensilla, aligned with two parallel rows of five spines. Moreover, two clusters of 14 chemosensilla were identified on the fifth tarsomere of female *Helicoverpa armigera* (Hübner) (Zhang et al., 2010).

Our electrophysiological recordings allowed for the differentiation of these sensilla into three functional types; TIα, TIβ, and TII showing different response spectra and sensitivity to the tested compounds. A clear correlation between the topology of the sensilla and function was found, where each functional type of sensillum was clustered, proximally (TIβ), medially (TII) or distally (TIα). The observed topological difference in function between gustatory sensilla is in line with that previously demonstrated for other insects, for example, on the antennae of the moth *Heliotis virescens* (Jørgensen et al., 2007) and on the labellum of the flies *Protophormia terraenovae* (Liscia et al., 1998) and *Drosophila melanogaster* (Hiroi et al., 2002). However, there are also examples where no such differences have been found, such as on the antennae of *S. littoralis* (Popescu et al., 2013) or on the tarsi of *M. private* (Calas et al., 2009).

Besides differences in functional response spectra between sensillum types, there was also a shift in amplitude of the neurons responding to NaCl and sugars between the two morphological types. While the largest spiking neuron of the TIα and TIβ sensilla, responded to sugar and the second largest to NaCl, the opposite pattern was found in the TII sensilla. Thus, the same stimulant seems be detected by GRNs generating different spike amplitudes in TI and TII sensilla. To our knowledge, the phenomenon of replacement of responding GRNs, associated with different types of gustatory sensilla of the same individual, has not been reported in other insects.

Individual sensilla, demonstrating similar topology, responded consistently to the tested compounds across most individuals tested, indicating that individual functional types of sensilla can provide specific information that affects behavioral decisions. In contrast, Blaney and Simmonds (1990) reported that the sensitivity of tarsal sensilla of female *S. littoralis* to sugars and the allelochemicals, azadirachtin and sinigrin, varied more between individuals than between sensilla of the same individuals. However, in their study, only one type of the tarsal gustatory sensilla (type-B, that corresponds to TI sensilla in our study) at the proximal and distal ends of the fifth tarsomere was tested. Similar to our results, tarsal gustatory sensilla, demonstrating identical morphology, of the moth *H. armigera* showed large individual differences in their response to different sugars, with topologically similar sensilla displaying the same response spectra between individuals (Zhang et al., 2010). It is possible that the three functional and morphological types of sensilla in *S. littoralis* process gustatory information differentially, and potentially play different roles in what behaviors they guide. Such a differentiation has been found in *D. melanogaster*, where two distinct morphological classes of sugar receptors on the fifth tarsomere provide information affecting different behaviors (Thoma et al., 2016); one class with axonal projections directly to the brain, which affect feeding initiation, and another class sending axonal projections to the thoracic ganglion, which influence suppression of locomotion.

A functional difference in the response to the two bitter compounds between the three sensillum types was found. Furthermore, the response to each of the two tested bitter compounds differed within each functional type. These differences indicate that there are different bitter receptors associated with different tarsal sensilla, but no specific GRN which would correspond to the presence of a specific deterrent cell, as suggested in other insects (Chapman, 2003). A specific deterrent cell would require uniformly and broadly tuned GRNs. Exemption to this, has however been described in *D. melanogaster* that has four different functional types of bitter sensing GRNs on the labellum (Weiss et al., 2011) and six on the foreleg (Ling et al., 2014). Even though our study was limited to two bitter compounds it indicated a clear difference in the response to bitter compounds between sensillum types. It is possible that a larger panel of bitter stimuli would have allowed for the identification of additional differences between functional sensillum types, also in *S. littoralis*. This is supported by a previous study on the polyphagous moth, *H. armigera*, where a high number of gustatory receptors, including a large number of bitter receptors, were identified (Xu et al., 2016). In contrast, more specialist Lepidopteran species tend to express an overall lower number of gustatory receptors (Xu et al., 2016), emphasizing the importance of bitter receptors in regulating host selection in specialists and generalists (McBride, 2007; McBride and Arguello, 2007). Thus, a polyphagous life style, such as that of *S. littoralis*, in which insects must be able to identify and evaluate a large number of different plant, may select for a higher diversity of gustatory receptors tuned to plant secondary compounds. The diversity of bitter receptors may also reflect that the response to bitter compounds can play a dual role for regulating behavioral output, and not only function to detect toxins or other harmful compounds. For example, studies on herbivore and pollinator insects have revealed that plant secondary metabolites, including alkaloids can elicit a range of behavioral responses from attraction to avoidance (Adler and Irwin, 2005; Manson et al., 2012).

Functional characterization of the tarsal sensilla of *S. littoralis* revealed that each functional type of tarsal sensillum housed four different classes of chemosensory neurons, as well as a mechanosensory neuron, which was activated when bending the sensillum. This is in line with a previous study on one type of tarsal gustatory sensilla of *S. littoralis*, showing activity to sugars, amino acids and secondary plant metabolites (Blaney and Simmonds, 1990). Among the three largest spiking neurons, clear dose-dependent responses to salt, sugars and bitter compounds was found, which has also been found for gustatory sensilla on the antennae of *S. littoralis* (Popescu et al., 2013). These characteristics are in line with that of gustatory sensilla among other insects (Städler, 1984; Chapman, 2003). Earlier studies on female butterflies *Pieris brassicae* showed that the gustatory sensilla of the fifth tarsomere include four GRNs; one neuron responding to secondary plant compounds, one to water and one to salt, whereas no response to sucrose was found (Ma and Schoonhoven, 1973). In our study, the responses of the N4-neuron, which exhibited the smallest spike amplitude, could not consistently be analyzed due to high background noise levels. In several orders of insects, including Lepidoptera, a corresponding neuron with the smallest spiking amplitude has been found to respond to water, or lower concentrations of sugars or amino acids (Schoonhoven and van Loon, 2002; Chapman, 2003). It is possible that also the N4-neuron in *S. littoralis* responds to these types of stimuli.

A difference in the sensitivity to sugars between different sensillum types was found, where the sugar sensitive neuron (N2) in the TII sensilla responded to sucrose, fructose, and glucose with approximately two times higher frequency than that of the TI sensilla (N1). The responses to the different tested sugars is similar with results from earlier studies on the gustatory sensilla on the tarsi, proboscis and larval maxillary styloconic sensilla of S. *littoralis* and other noctuid moths (Blaney and Simmonds, 1988, 1990). Sugar sensitive GRNs on the tarsi are common among insects and have, for example, been found in bees (de Brito Sanchez et al., 2014) and in flies, such as, blowflies (Liscia et al., 1998) and *D. melanogaster* (Hiroi et al., 2002; Ling et al., 2014). Among the three tested sugars, sucrose elicited the highest firing rate, which is in line with other studies on moths (Blaney and Simmonds, 1988; Zhang et al., 2010). Different sensitivity to sugars may depend on the molecular structure of the tested sugars. A relationship between the chemical structure of sugar and the sensitivity of the tarsal chemoreceptors has also been found in the butterfly *Pieris rapae* (Kusano and Sato, 1980) and in some moths (Ramaswamy, 1987; Zhang et al., 2010). Such differences in sensitivity suggest that moths could discriminate between different relevant sugars through specific coding mechanisms where different sugars interact with different receptor proteins expressed by the same receptor neurons (Zhang et al., 2010), which has been found in labellar GRNs of flies (Shimada, 1975; Hiroi et al., 2002).

Both inhibition and excitation of the activity of neurons responding to sugars and salt was found when stimulating with the bitter compounds, caffeine and quinine. Furthermore, the firing frequency of the sugar sensitive GRN was inhibited with increasing concentrations of caffeine, when presented as a mixture, which is in line with a previous study on larvae of *S. littoralis* (Simmonds et al., 1990). Interactions between secondary compounds and sugars have also been found in adult *S. littoralis* and other Lepidopteran species, such as *S. frugiperda, H. armigera* and *H. virescens* (Blaney and Simmonds, 1990) and *H. virescens* (Jørgensen et al., 2007), which directly may affect the behavior of the insect, as shown for the blowfly, *P. terraenovae* (Liscia and Solari, 2000). This indicates that bitter compounds can affect behavioral output both by signaling unsuitability of the substrate, but also by inhibiting the responses to stimulants, such as sugars. The latter mechanism, is independent of the activity of the GRN responding to bitter compounds (French et al., 2015), and could be a common strategy to regulate feeding (Freeman and Dahanukar, 2015). It also shows that interaction between different types of stimuli can occur already at the peripheral level. Lastly, the electrolyte, 10 mM NaCl, was not considered in interaction studies and has previously not been shown to affect the response of the two tested compounds.

Our study shows a morphological and functional differentiation in the gustatory sensilla on the tarsi of *S. littoralis*. This provides the insect a basis for detecting relevant sensory cues when alighting on a host plant. Previous work from our group has shown that olfactory receptors on the antennae are important for phenotypic plasticity and adaptation to new environments (Lhomme et al., 2018). These experiments also indicate that gustatory information is involved in the mechanism driving this plasticity. More knowledge is needed to find the role of the gustatory stimuli in this plasticity and how they interact with olfactory stimuli during host plant selection in *S. littoralis*.

## Author Contributions

MS, PA, and RI designed the study and wrote the manuscript in a joint effort. MS performed the experiments and analysis under the supervision of PA and RI. All authors contributed to the outline of this work.

## Conflict of Interest Statement

The authors declare that the research was conducted in the absence of any commercial or financial relationships that could be construed as a potential conflict of interest.
